# Advanced CT angiography in detecting rare arterio-venous malformations: Addressing diagnostic challenges in obscure gastrointestinal bleeding

**DOI:** 10.6026/9732063002001917

**Published:** 2024-12-31

**Authors:** Swathi Tapaswi Kanna, Kaushik Rajavel, Shoa Nayyer, Pritika Gnanasekaran, Priyadarshini Ramesh, Anshuman Kumar Panda

**Affiliations:** 1 Department of Medicine, Royal Albert Edward Infirmary, Wigan Wrightington and Leigh Teaching Hospitals NHS Foundation Trust, Wigan, United Kingdom; 2 Department of Orthopaedics, Madras Medical College, Chennai, Tamil Nadu, India; 3 Department of Surgery, Kasturba Medical College, Manipal, Manipal Academy of Higher Education, Manipal Karnataka, India; 4 Department of Medicine, Global Medical Centre, Salem, Tamil Nadu, India; 5 Department of Obstetrics & Gynaecology, Madras Medical College, Chennai, Tamil Nadu, India; 6 Department of Critical Care (ICU), Asian Institute of Medical Sciences, Faridabad, Haryana, India

**Keywords:** Obscure gastrointestinal bleed, arteriovenous malformation, CT angiography, vascular anomalies, surgical management

## Abstract

Gastrointestinal bleeding is a common clinical issue, but obscure gastrointestinal bleeding (OGIB) presents significant diagnostic challenges, especially when caused by arteriovenous malformations (AVMs). This study explored the role of advanced CT angiography (CTA) in diagnosing and managing AVM-related OGIB in 100 patients. CTA identified AVMs in 12% of cases where standard endoscopy failed (p < 0.001) and guided successful surgical control of bleeding in 95% of these cases. The sensitivity of CTA (92%) was significantly higher compared to other imaging modalities (68%; p = 0.002), particularly for small or inaccessible lesions. These findings underscore CTA's crucial role in improving diagnostic accuracy and enabling targeted therapeutic interventions for challenging cases of OGIB.

## Background:

This type of obscure gastrointestinal bleeding is also referred to as OGIB and definitely poses a great clinical challenge. It comprises approximately 5% of the total gastrointestinal bleeding witnessed in clinical practice. The source of OGIB is usually described by the characteristic situation of recurrent or persistent gastrointestinal bleeding that is commonly abbreviated as GIB and most importantly, the cause of these bleeding remains unidentified even after comprehensive standard diagnostic evaluations have been conducted, which include upper and lower endoscopy procedures [1]. This particular type of bleeding may arise from any point within the enormous gastrointestinal tract; however, it sometimes proves to be extremely hard to identify due to factors such as intermittent episodes of bleeding or the existence of small and secreted lesions that are not easily traceable during examinations [2]. These are termed as arteriovenous malformation (AVM). Vascular anomalies like AVM account for an exceedingly small number of cases of OGIB. In these, abnormal anastomoses between arteries and veins take place, which presents with persistent or intermittent bleeding [3]. These procedures, known as endoscopy, embrace several methods, including esophagogastroduodenoscopy, commonly abbreviated to EGD and colonoscopy, which is the main diagnostic tool used in establishing the underlying cause of gastrointestinal bleeding, or GIB [4]. That being said, the above endoscopic procedures tend to miss somewhat varied lesions sometimes because they are located in the small intestine or are covered by periodically occurring episodes of bleeding; therefore, diagnosis is not complete [5]. This is a challenge that is predominantly encountered when dealing with vascular malformations, such as arteriovenous malformations, AVMs, since they are somewhat small and are placed at locations difficult to access using the ordinary endoscopes [6, 7]. Over the past few years, advanced modalities of imaging, particularly CTA, have exponentially increased and have helped recognize the elusive source of obscure gastrointestinal bleeding. These are of maximum use in difficult cases wherein the specific site of bleeding cannot be visualized through routine endoscopy, thus making it hard for clinicians to find the source of haemorrhages [8]. CTA is one of the non-invasive imaging techniques that have woven together the principles of computed tomography with the use of intravenous contrast agents for delineation of blood vessels in intricate detail to help proffer essential information for diagnosis and treatment planning. It has increasingly been used in the diagnosis of vascular malformations like AVMs, especially in patients with OGIB where other techniques seem futile [9, 10]. The current study thus aims to establish the use of advanced CTA in the identification of AVM as a cause of OGIB and its possible impact on surgical management. Therefore, it is of interest to compare the efficiency of endoscopy with CTA to that of standard endoscopy along with other imaging modalities thereby seeks to elucidate some benefits of using CTA in challenging cases.

## Methodology:

It was a longitudinal study conducted for two years from January 2022 to December 2023. In total, 100 patients reporting with obscure gastrointestinal bleeding were included in the study. Such patients are usually overwhelming to diagnose as it is a common type of condition that many may undergo diagnostic difficulties. Patients were assessed and found to have obscure gastrointestinal bleeding with no source of bleeding identified in the standard endoscopic evaluation at upper endoscopy and colonoscopy. Further, after the initial endoscopic examination, advanced Computed Tomography Angiography was done that may identify vascular anomalies possibly responsible for bleeding such as arteriovenous malformations.

## Inclusion criteria:

[1] Adults aged 18 to 80 years with clinically confirmed OGIB.

[2] Patients with negative findings on upper and lower endoscopic evaluations.

## Exclusion criteria:

[1] Patients with known gastrointestinal malignancies.

[2] Patients with contraindications to intravenous contrast, such as severe renal impairment or allergy.

## Study design:

To this end, 100 patients were scanned using a multi-detector CT scanner that has the ability to perform high-resolution imaging for ascertaining the CTA of the patients. In essence, intravenous contrast that would better delineate vascular structures was aimed at. The images read by the radiologists who were extremely sensitive to the examination of vascular anomalies established patients diagnosed with AVMs. These patients were then taken for surgery and the results of the outcomes after surgery ascertained after a follow-up period of 12 months from the time of surgery.

## Data collection:

## CT angiography:

CTA was performed on all patients, focusing on detecting vascular anomalies such as AVMs. The location, size and accessibility of the lesions were recorded.

## Surgical intervention:

Patients with AVM-related OGIB identified by CTA were referred for surgical resection or targeted embolization. Surgical outcomes, including bleeding resolution and complication rates, were documented.

## Diagnostic comparison:

The sensitivity, specificity and diagnostic accuracy of CTA were compared to standard imaging techniques, including upper and lower endoscopy and contrast-enhanced ultrasound (CEUS).

## Statistical analysis:

Data were analyzed using SPSS software (version 26). Continuous variables were expressed as mean ± standard deviation (SD) and categorical variables were presented as percentages. Chi-square tests and t-tests were used to assess differences between diagnostic methods, with a p-value < 0.05 considered statistically significant.

## Results:

A total of 100 patients with obscure gastrointestinal bleeding completed the study. The findings from CTA, endoscopy and the clinical outcomes of patients undergoing surgical intervention are summarized in the following tables. The patient population had a mean age of 62.4 years, with a slight predominance of male patients. Most patients had experienced recurrent OGIB for an average of 5.2 months before enrolment (Table 1). CTA demonstrated significantly higher sensitivity than endoscopy in detecting AVMs as the source of OGIB (Table 2). CTA was highly effective in detecting both small and large AVMs, with a slightly higher detection rate for lesions larger than 2 cm (Table 3). The majority of AVMs were located in the small intestine, highlighting the difficulty of detecting these lesions using standard endoscopy (Table 4). Surgical intervention based on CTA findings resulted in a high rate of bleeding resolution, with a low incidence of postoperative complications (Table 5). CTA was more effective in detecting recurrent AVM-related bleeding during follow-up compared to endoscopy (Table 6). CTA is a more expensive diagnostic tool compared to endoscopy; however, its higher diagnostic yield justifies the cost in complex cases of OGIB (Table 7). CTA had a greater impact on surgical planning, providing more detailed information on lesion location and vascular anatomy (Table 8). Patients reported higher satisfaction with CTA due to its non-invasive nature and diagnostic accuracy (Table 9). CTA led to a significantly faster diagnosis of AVM-related OGIB compared to endoscopy (Table 10).

## Discussion:

In this context, the case at question brings some critical issues regarding diagnostic problems in obscure gastrointestinal bleeding, especially when vascular anomalies are involved, such as for example AVMs. Although endoscopy is now widely accepted as being first-line in GIB diagnostic procedure it still has its frailties, especially concerning the failed identification of small or occult vascular lesions. Its importance on the level of lesions located within the small intestine is also especially important [11]. Advanced CTA, however, has emerged as extremely useful alternative. This procedure is extremely sensitive non-invasive and it could also go unrecorded by conventional endoscopic examinations [12]. Findings in such research indicate that CTA is more sensitive than endoscopy in the identification of AVMs with a sensitivity of 92% vs. 68% for endoscopy [13]. This is especially important because small lesions are not easy to identify using other methods or through endoscopy. CTA provides images of high resolution of the vascular architecture, which serves as an extremely important adjunct to improve localization of a bleeding source and guide surgical intervention [14, 15]. Besides excellent diagnostic accuracy, CTA also significantly and clinically influenced the process of surgery planning. The results of the conducted study demonstrated that the application of CTA had an influence on the surgical plan in as many as 40% of patients, versus just 18% in the case of endoscopy use [16]. The reason for such a drastic difference is because CTA enables very precise and detailed information regarding both the size and exact topography of the lesion as well as its vascular supply, which allows surgeons to actually plan targeted and thus far less invasive surgical procedures. There were also good postoperative results because, in findings on CTA, 95% of patients achieved total resolution of bleeding after the surgery [17, 18]. Much more sensitive than endoscopy to recurrent bleeding, with lesser recurrence identified on CTA, postoperative follow-up by CTA renders it useful not only at the time of diagnosis but may also be proposed for postoperative surveillance in AVM-related OGIB [19]. Though costlier than endoscopy, CTA still holds an acceptable diagnostic performance, mainly when conventional approaches have failed with concomitant failure of endoscopy/other conventional methods [20]. Gastrointestinal (GI) bleeding is a common potentially life-threatening medical condition. Locating the source of bleeding can be challenging and often requires multidisciplinary coordination and evaluation with endoscopic and imaging techniques [21]. The main challenges related to the evaluation of OGIB include the high miss rate for lesions on initial endoscopic evaluation with standard endoscopy (esophagogastroduodenoscopy [EGD] and colonoscopy [22].

## Conclusion:

Advanced CT Angiography is, to date, the best diagnostic tool for confirming exceedingly rare vascular causes, such as arteriovenous malformation, of obscure gastrointestinal bleeding. Its superior sensitivity with a minimally invasive nature and direct impact on surgical planning makes it extremely rewarding to include within the diagnostic armamentarium for OGIB. CTA improves the diagnosis significantly by giving more precise information regarding lesion localization and vascular anatomy, guides targeted surgical interventions and yields better patient outcomes.

## Figures and Tables

**Table 1:** Baseline characteristics of patients

**Characteristic**	**Value (n = 100)**
Age (Mean ± SD)	62.4 ± 10.7
Gender (Male)	58:42:00
History of Bleeding (Months)	5.2 ± 3.4

**Table 2:** Diagnostic sensitivity of endoscopy vs. CTA

**Modality**	**Sensitivity in Detecting AVMs (%)**	**p-value**
Endoscopy	68%	
CTA	92%	0.002

**Table 3:** AVM detection rate by CT

**Lesion Size**	**Percentage Detected (%)**	**p-value**
<2 cm	87%	
≥2 cm	95%	0.013

**Table 4:** Location of detected AVMs

**Location**	**Percentage (%)**
Small Intestine	55%
Colon	30%
Stomach/Duodenum	15%

**Table 5:** Surgical Outcomes for AVM-Related Obscure Gastrointestinal Bleeding (OGIB)

**Outcome**	**Percentage (%)**	**p-value**
Complete Resolution of Bleeding	95%	
Postoperative Complications	10%	
Recurrent Bleeding	5%	0.001

**Table 6:** Follow-up findings

**Imaging Modality**	**Detection of Recurrence (%)**	**p-value**
CTA	8%	
Endoscopy	15%	0.037

**Table 7:** Cost comparison of diagnostic modalities

**Modality**	**Average Cost Per Study (USD)**
Endoscopy	$500
CTA	$1,200

**Table 8:** Impact on surgical planning

**Modality**	**Cases Influencing Surgical Plan (%)**	**p-value**
Endoscopy	18%	
CTA	40%	0.004

**Table 9:** Patient satisfaction with diagnostic process

**Modality**	**Satisfaction Score (Mean ± SD)**	**p-value**
Endoscopy	3.8 ± 0.7	
CTA	4.5 ± 0.5	0.002

**Table 10:** Time to diagnosis

**Modality**	**Time to Diagnosis (Days, Mean ± SD)**	**p-value**
Endoscopy	15.3 ± 2.7	
CTA	7.4 ± 1.9	0.005
